# The interplay of plasticity and adaptation in neural circuits: a generative model

**DOI:** 10.3389/fnsyn.2014.00026

**Published:** 2014-10-30

**Authors:** Alberto Bernacchia

**Affiliations:** School of Engineering and Science, Jacobs University BremenBremen, Germany

**Keywords:** synaptic plasticity, sensory adaptation, dynamical systems, attractor model, generative model, Bayesian inference

## Abstract

Multiple neural and synaptic phenomena take place in the brain. They operate over a broad range of timescales, and the consequences of their interplay are still unclear. In this work, I study a computational model of a recurrent neural network in which two dynamic processes take place: sensory adaptation and synaptic plasticity. Both phenomena are ubiquitous in the brain, but their dynamic interplay has not been investigated. I show that when both processes are included, the neural circuit is able to perform a specific computation: it becomes a generative model for certain distributions of input stimuli. The neural circuit is able to generate spontaneous patterns of activity that reproduce exactly the probability distribution of experienced stimuli. In particular, the landscape of the phase space includes a large number of stable states (attractors) that sample precisely this prior distribution. This work demonstrates that the interplay between distinct dynamical processes gives rise to useful computation, and proposes a framework in which neural circuit models for Bayesian inference may be developed in the future.

## 1. Introduction

The main goal of Computational Neuroscience is to uncover the kinds of computation implemented by neurons and neural circuits, and to identify the biological mechanisms underlying these computations. Numerous types of computation have been described and have been associated with the dynamics of different neural and synaptic processes (Herz et al., 2006; Abbott, 2008; Gerstner et al., 2012; Tetzlaff et al., 2012). Among the numerous biological phenomena observed in the brain, sensory adaptation and synaptic plasticity stand out as two of the most studied, since they are observed ubiquitously across most brain regions and animal species. Both phenomena give rise to specific types of computation, but the functional implications of their interaction remain unclear.

Synaptic plasticity is the change in strength of the interaction between neurons, and is believed to control the change in behavior of a subject following its experience of the external world. Synaptic plasticity takes multiple forms (Abbott and Nelson, 2000; Feldman, 2009), of which the most studied is Hebbian plasticity (Bi and Poo, 2001; Caporale and Dan, 2008). Different types of plasticity are believed to underlie a broad range of functions, including: memory formation and storage (Martin et al., 2000; Lamprecht and LeDoux, 2004; Seung, 2009), nervous system development (Katz and Shatz, 1996; Miller, 1996; Sanes and Lichtman, 1999; Song and Abbott, 2001), recovery after brain injury (Buonomano and Merzenich, 1998; Feldman and Brecht, 2005), classical conditioning (Wickens et al., 2003; Calabresi et al., 2007; Surmeier et al., 2009; Pawlak et al., 2010; Gallistel and Matzel, 2013), operant conditioning (Seung, 2003; Montague et al., 2004; Daw and Doya, 2006; Doya, 2007; Soltani and Wang, 2008), spatial navigation (Blum and Abbott, 1996; Mehta et al., 2002), efficient coding of sensory stimuli (Toyoizumi et al., 2005; Savin et al., 2010; Bernacchia and Wang, 2013), homeostatic regulation of neuronal excitability (Royer and Paré, 2003; Turrigiano and Nelson, 2004; Williams et al., 2013), sound localization (Gerstner et al., 1996) and production of behavioral sequences (Fiete et al., 2010).

Sensory adaptation is the change in responsiveness of a neuron to a given input, and is believed to control the change in perception of a stimulus, even if the stimulus maintains constant physical attributes (Webster, 2011). The main effect of adaptation on neural activity is to shift the response function depending on the adapting stimulus (Kohn, 2007; Rieke and Rudd, 2009). This effect has been observed in a broad range of species, sensory modalities and stimulus variables, including: luminance (Sakmann and Creutzfeldt, 1969; Shapley and Enroth-Cugell, 1984), contrast (Ohzawa et al., 1985; Smirnakis et al., 1997), edge orientation (Müller et al., 1999; Dragoi et al., 2000), direction of motion (Kohn and Movshon, 2004), motion speed (Brenner et al., 2000; Krekelberg et al., 2006) and sound level (Dean et al., 2005). In addition to the shift in tuning, the gain of neural response changes depending on the stimulus variance (Fairhall et al., 2001; Borst et al., 2005; Nagel and Doupe, 2006; Maravall et al., 2007). One hypothesized function of sensory adaptation is the efficiency of coding: the statistics of input stimuli can vary widely and must be encoded by neurons with limited dynamic range; centering the neural response around the mean input prevents saturation and determines optimal discrimination (Laughlin, 1981; Wainwright, 1999; Machens et al., 2005; Clifford et al., 2007; Schwartz et al., 2007; Wark et al., 2007).

I simulate and analyze a computational model of a recurrent neural circuit, and I show that when both sensory adaptation and synaptic plasticity are included in the model, the neural circuit is endowed with a specific type of computation: it becomes a generative model of the input stimuli. Generative models provide a solution to a broad range of problems in machine learning (Hinton, 2007, 2010; Barra et al., 2012), and have been proposed as candidate models of perception, learning and Bayesian inference in real brains (Fiser et al., 2010; Clark, 2013). In the model presented here, the spontaneous dynamics of neural activity lingers on a subset of specific neural patterns which correspond to the neural patterns that have been driven by sensory stimuli. In particular, the likelihood of observing a given neural pattern is equal to the frequency with which the corresponding stimulus has been previously experienced. Formally speaking, the model dynamics displays a large number of attractors which sample exactly the probability distribution of input stimuli. In the limit of an infinite number of neurons, I show that the dynamics converges to a continuous (line) attractor.

Neurons and synapses are modeled as binary variables (Hopfield, 1982; Tsodyks, 1990), therefore the model is not biologically realistic. In particular, it does not include separate populations of excitatory and inhibitory neurons and does not account for a range of dynamical regimes observed in the brain, such as the asynchronous and irregular spiking activity of cortical neurons. Also, the network operates in two distinct phases: (1) a stimulus driven regime in which plasticity and adaptation occur and internal dynamics is turned off, and (2) a spontaneous regime in which the stable states of the dynamics are probed in absence of stimulus, plasticity and adaptation. However, the model is very simple to simulate and analyze despite the inclusion of multiple mechanisms. The present work is limited to univariate distributions of input stimuli.

## 2. Materials and methods

The neural circuit implemented in this work is a variant of a model studied in Bernacchia and Amit (2007), with the additional inclusion of adaptation. I consider a neural circuit with a total number of neurons equal to *N*, labeled by the index *i* = 1, …, *N*. The total current afferent to neuron *i* at time *t* is the sum of the external current, due to the input stimulus, and the internal current, due to the local recurrent connections within the neural circuit:

(1) $$I_{i}(t)=I_{i}^{ext}(t)+I_{i}^{int}(t)$$

The activity of neuron *i* upon receiving current *I_i_* is equal to

(2) $$x_{i}(t+\Delta t)=\text{sign}\left(I_{i}(t)\right)$$

where Δ*t* is the time step used in simulations.

For simplicity, I consider two exclusive scenarios, in which either of the two types of currents dominate: When a stimulus is presented, the internal current is set to zero, therefore the external current dominates (“stimulus-driven” stage); when a stimulus is absent, the external current is set to zero and the internal current dominates (“spontaneous” stage). The case in which both types of current are simultaneously contributing was studied in Bernacchia and Amit (2007) (in absence of adaptation). The parameter Δ*t* reflects the time constant of the update of a neuron's activity, and is set to 10 ms during the spontaneous stage. During the stimulus-driven stage, a sequence of stimuli is presented, the activity is instantaneously enforced by the stimulus. Therefore, the activity is constant as long as the stimulus is constant, and changes immediately following transitions between subsequent stimuli. For simplicity, I simulate one time step for each stimulus, by setting the time step equal to one “trial,” Δ*t* = 1. This value of Δ*t* is used only for convenience of numerical integration, and is not related to any biological timescale. A total of *T* number of trials is simulated (a sequence of *T* stimuli) in one simulation, *t* = 1, …, *T*.

The stimulus identity is labeled by α, varying in the closed interval of real numbers α ∈ (0,1), and the stimulus presented at time *t* is denoted as α(*t*). The stimulus value α(*t*) at each time step is drawn at random from a probability distribution *P*(α). The external current afferent to neuron *i* depends on how that neuron is tuned to the stimulus, which is summarized by its “tuning curve.” I consider two types of tuning curves in different simulations, one monotonic (sigmoidal), and one periodic (sine), given by the following simple formulas

(3) $$I_{i}^{ext}(t)=\tanh(\beta [\alpha (t)-\mu_{i}])$$

(4) $$I_{i}^{ext}(t)=\sin(2\pi [\alpha (t)-\mu_{i}])$$

**Figure 1:**
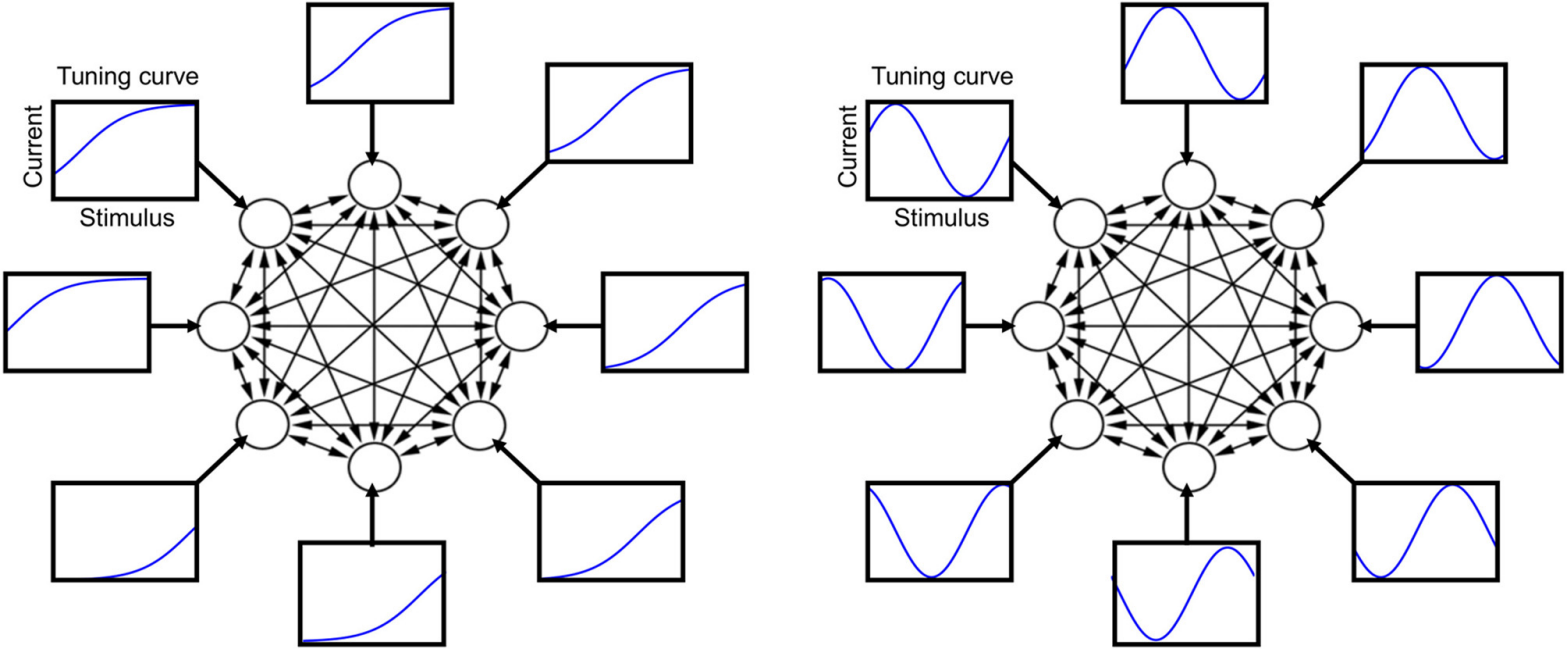
**Schematic illustration of the neural circuit model with its tuning curves and recurrent connections**. Each circle represents one neuron and each arrow a synaptic connection. Each rectangle shows a tuning curve for one neuron, namely the external current afferent to that neuron plotted as a function of the stimulus value. **Left**: sigmoidal tuning curves. **Right**: periodic tuning curves.

An illustration of the tuning curves is presented in Figure 1. I define μ_*i*_ as the “tuning offset”: different neurons have different offsets, but the same shape of the tuning curve. The results of most simulation are shown for the sigmoidal tuning curve (3), but very similar results have been obtained for a periodic tuning curve (4) (see Appendix). The parameter β is positive, and its specific value is irrelevant, since the neuron output is binary, given by Equation (2), and the internal current is zero during the stimulus. In a given simulation, the probability distribution *P*(α) is taken from a parametric family (see e.g., **Figure 3**), and different parameters are drawn at random in different simulations (the distribution equals the square of a Fourier series with random coefficients truncated at five terms).

The internal current is the sum of activity *x_j_* of pre-synaptic neurons weighted by the synaptic matrix *J_ij_*, namely

(5) $$I_{i}^{int}(t)=\frac{1}{2N}\sum_{j}J_{ij}(t)x_{j}(t)$$

Synaptic weights have binary values, *J_ij_* = ±1, except for the self-couplings that are set to zero *J_ii_* = 0. Synaptic strengths are initialized at random, +1 and −1 with equal probability. The synaptic plasticity rule is Hebbian, meaning that it follows the correlation of the pre and post-synaptic neuron (the product *x_i_x_j_*). Synaptic weights are updated at random at each time step according to the following transition probabilities. The probability of potentiation from time *t* to time *t* + Δ*t* is the probability of the transition from *J_ij_*(*t*) = −1 to *J_ij_*(*t* + Δ*t*) = +1, and is defined as

(6) $$w_{+}=\frac{1+x_{i}x_{j}}{2\tau }$$

Conversely, the probability of depression from time *t* to time *t* + Δ*t* is the probability of the transition from *J_ij_*(*t*) = +1 to *J_ij_*(*t* + Δ*t*) = −1, and is defined as

(7) $$w_{-}=\frac{1-x_{i}x_{j}}{2\tau }$$

Therefore, if *x_i_* and *x_j_* are different, there is a probability 1/τ of synaptic depression, while if they are equal there is a probability 1/τ of synaptic potentiation. The time constant τ represents the average number of time steps necessary to observe a transition (in units of Δ*t*). Note that this synaptic plasticity rule is symmetric, namely the same transition probabilities apply to *J_ij_* and *J_ji_*. In order to reduce the effect of finite-size noise, symmetry of synaptic weights is enforced at each transition by updating half of the synapses and setting *J_ij_* = *J_ji_*. This enforcement does not change the qualitative behavior of the model.

**Figure 2:**
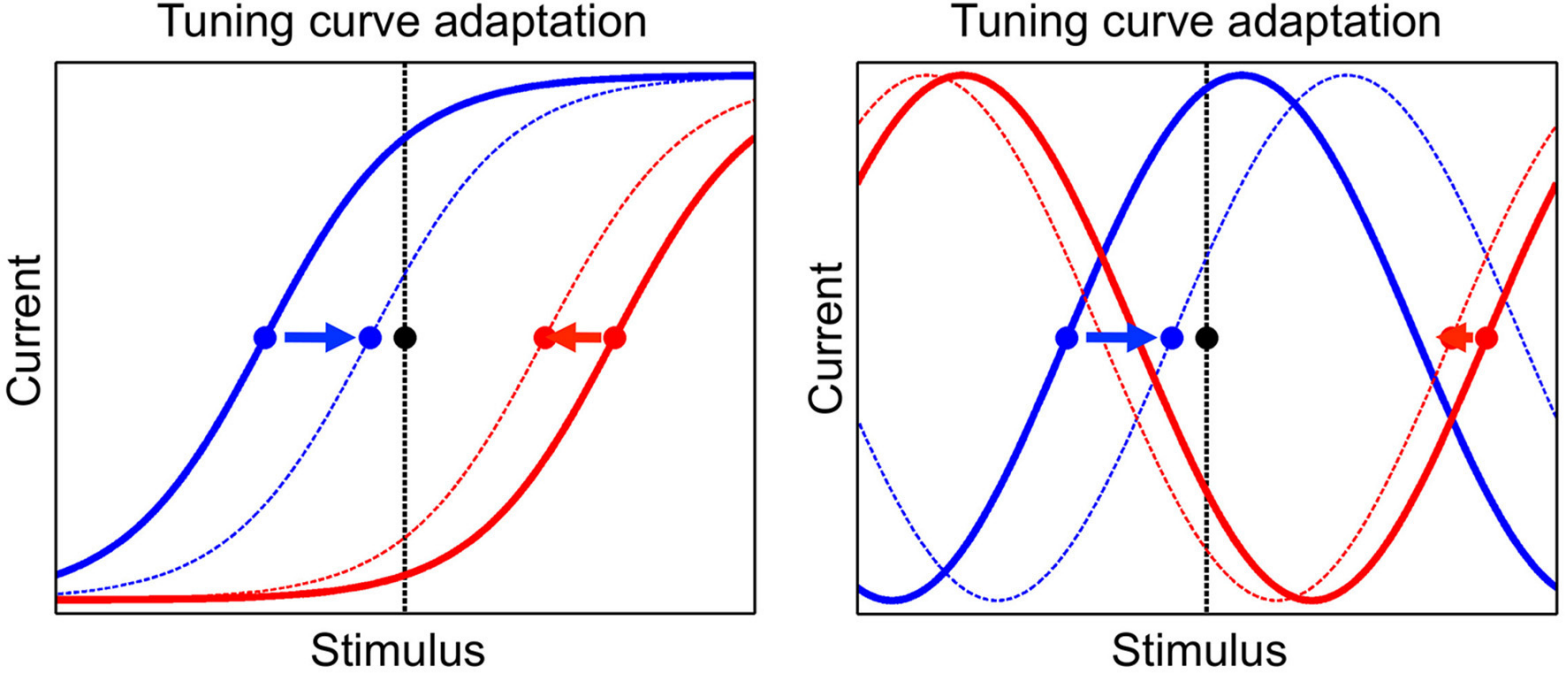
**The adaptation mechanism, by which tuning curves of neurons are modified according to the presented stimulus**. Tuning curves of two neurons are shown, one neuron in blue and the other one in red, before and after adaptation (full and dashed line, respectively). The presented stimulus is indicated by the black dot and the vertical black line. The tuning offsets of the two neurons are shown by the blue and red dots. Tuning offsets are attracted by the stimulus, as shown by the arrows. **Left**: sigmoidal tuning curves. **Right**: periodic tuning curves.

The tuning curve of a neuron is modified during presentation of stimuli, as a consequence of adaptation. We implement a phenomenon known as adaptation to the mean, which is ubiquitously observed in a wide range of species, sensory modalities and stimulus variables (Wark et al., 2007; Rieke and Rudd, 2009). In particular, the presentation of a given stimulus determines a change in the tuning offsets of neurons such that they tend to converge toward that stimulus. An illustration of this dynamics is shown in Figure 2. The tuning offset is a function time, μ_*i*_(*t*), and changes according to

(8) $$\mu_{i}(t+\Delta t)=\mu_{i}(t)+\frac{1}{\tau }\left[\mu_{i}^{0}-\Theta (\mu_{i}(t)-\alpha (t))\right]$$

where τ is the timescale of adaptation in units of Δ*t* (τ is chosen equal to the timescale of plasticity), α(*t*) is the stimulus presented at time *t*, and Θ is the step function. The initial values of the tuning offsets μ_*i*_(0) = μ^0^_*i*_ are chosen to span uniformly the interval (0,1), namely

(9) $$\mu_{i}^{0}=\frac{i-1/2}{N}\;i=1,\ldots ,N$$

The dynamics of tuning offsets, Equation (8), implies that they are attracted by the presented stimulus, more so if they are closer to it (see Figure 2). For convenience, tuning offsets are reordered at each time step such that μ_1_ < … < μ_*N*_. Namely, after each update by Equation (8), if μ_*i*_ < μ*_j_* for *i* > *j* then μ_*i*_ → μ_*j*_ and μ_*j*_ → μ*_i_* (no other neural parameters are permuted in this step). Learning works without the permutation step but simplifies its implementation.

The spontaneous dynamics of the network is tested 10 times in each simulation, at fixed intervals of T/10 trials, referred to as 10 sessions. In each session of the spontaneous stage, external current, plasticity and adaptation are turned off, and the internal current is turned on. Recurrent neural dynamics is simulated with a time step Δ*t* that is different from the one used during the stimulus-driven stage. In the stimulus driven stage, Δ*t* reflects the presentation of a stimulus, which may occur on a time interval of a few seconds, while Δ*t* in the spontaneous stage reflects the fast interaction between neurons through the internal currents, of the order of tens of milliseconds. This internal dynamics is implemented, by running Equations (2), (5), until the network reaches a stable fixed point, when neural activity does not change from one time step to the next. Then, this stable state is recorded and the stimulus driven dynamics is resumed until the next session.

Across the 10 sessions of one simulation, the stable state may change as a consequence of the changes in synapses occurred during the stimulus-driven stage. I show in the Appendix that the stable state must have a specific form that depends on a single parameter ν (see Appendix: “The spontaneous dynamics of neurons”). In general, this function is denoted by ξ(ν, μ_*i*_) and, for a sigmoidal tuning curve, that is equal to

(10) $$x_{i}=\xi (\mu_{i},\nu )=\text{sign}(\nu -\mu_{i})$$

where ν is defined as the “retrieved” pattern and is sufficient to identify the entire network state. This form implies that the spontaneous state is equal to a pattern that would be obtained in presence of stimulus α = ν. In order to explore the possibility of the existence of multiple stable states, I run several simulations each one with a different initial condition, varying across the possible values of retrieved patterns.

The model has three parameters, *N*, *T*, and τ, fixed in each simulation. I used *N* = 1000 in most simulations, with a few simulations implementing *N* = 2000, 4000, 8000, and 16000. Three values of *T* used in simulations are *T* = 1000, *T* = 10000, and *T* = 100000. Three values of τ used in simulations are τ = 100, τ = 1000, and τ = 10000.

## 3. Results

A simulation of neural circuit dynamics is divided in two separate stages: a stimulus-driven stage and a spontaneous stage. During the stimulus-driven stage, sensory stimuli are presented and the external currents dominates over the internal currents. The response of a neuron to external stimuli is characterized by the tuning curve of that neuron (illustrated in Figure 1). During the spontaneous stage, there is no sensory stimulus and the neural circuit activates autonomously according to its internal currents. Synaptic plasticity and sensory adaptation occur during the stimulus-driven stage. Synaptic plasticity is implemented by a simple Hebbian rule, while sensory adaptation is implemented by modifying the tuning curve of neurons (illustrated in Figure 2). In each simulation, 10 sessions of stimulus-driven dynamics alternate with 10 sessions of spontaneous dynamics (see Materials and Methods for details).

Synaptic strengths are initialized at random, and they start switching as a result of plasticity, depending on the neural patterns of activity enforced by the presentation of stimuli. Upon presentation of a given stimulus, synaptic plasticity tends to make the corresponding neural activity pattern more stable, because of the Hebbian rule. A series of different stimuli are subsequently presented, determining a series of corresponding neural activity patterns. Therefore, because of the presentation of multiple stimuli, each pattern competes with other patterns for switching synapses in its own favor.

However, neural activity patterns not only compete but they also cooperate. Since the tuning curves of neurons are smooth functions of the stimulus (Figure 1), two similar values of the stimulus corresponds to two neural activity patterns that are also similar. Therefore, neural activity patterns are correlated, and two similar patterns collaborate in switching synapses toward stabilizing both of them. The resolution of this competition-cooperation trade-off depends on the distribution of input stimuli. If a subset of nearby stimuli is presented more often than other stimuli, the corresponding neural activity patterns will stabilize at the expense of others.

In order to determine which neural activity patterns stabilize as a consequence of synaptic plasticity, I measure the stable fixed points of the spontaneous dynamics, also referred to as “attractors” or “stable states.” At ten regular intervals (sessions) in each simulation, the stream of external stimuli is interrupted and the spontaneous dynamics is tested in presence of the internal currents only. This dynamics runs until the network reaches a stable fixed point, a neural activity pattern that does not change unless the system is perturbed. In each session, the spontaneous dynamics runs multiple times, with different initial conditions, to test for multiple stable states. After recording all the stable states, the stimulus-driven dynamics is resumed until the next session. The process is repeated for the 10 sessions of each simulation (see Materials and Methods).

The neural activity pattern corresponding to a given stable state is summarized by a single parameter, the “retrieved pattern.” This corresponds to the stimulus that, when presented, elicits exactly that neural pattern of activity. During the spontaneous dynamics there is no presentation of any stimulus, nevertheless the stable state is equivalent to the pattern elicited by that stimulus. The fact that the spontaneous dynamics reproduces the activity corresponding to a stimulus implies that synaptic plasticity has previously worked toward stabilizing that stimulus. I show in the Appendix (section: “The spontaneous dynamics of neurons”) that a spontaneous state is equal to a stimulus pattern provided that stimulus-driven synaptic plasticity has occurred for a time long enough.

**Figure 3:**
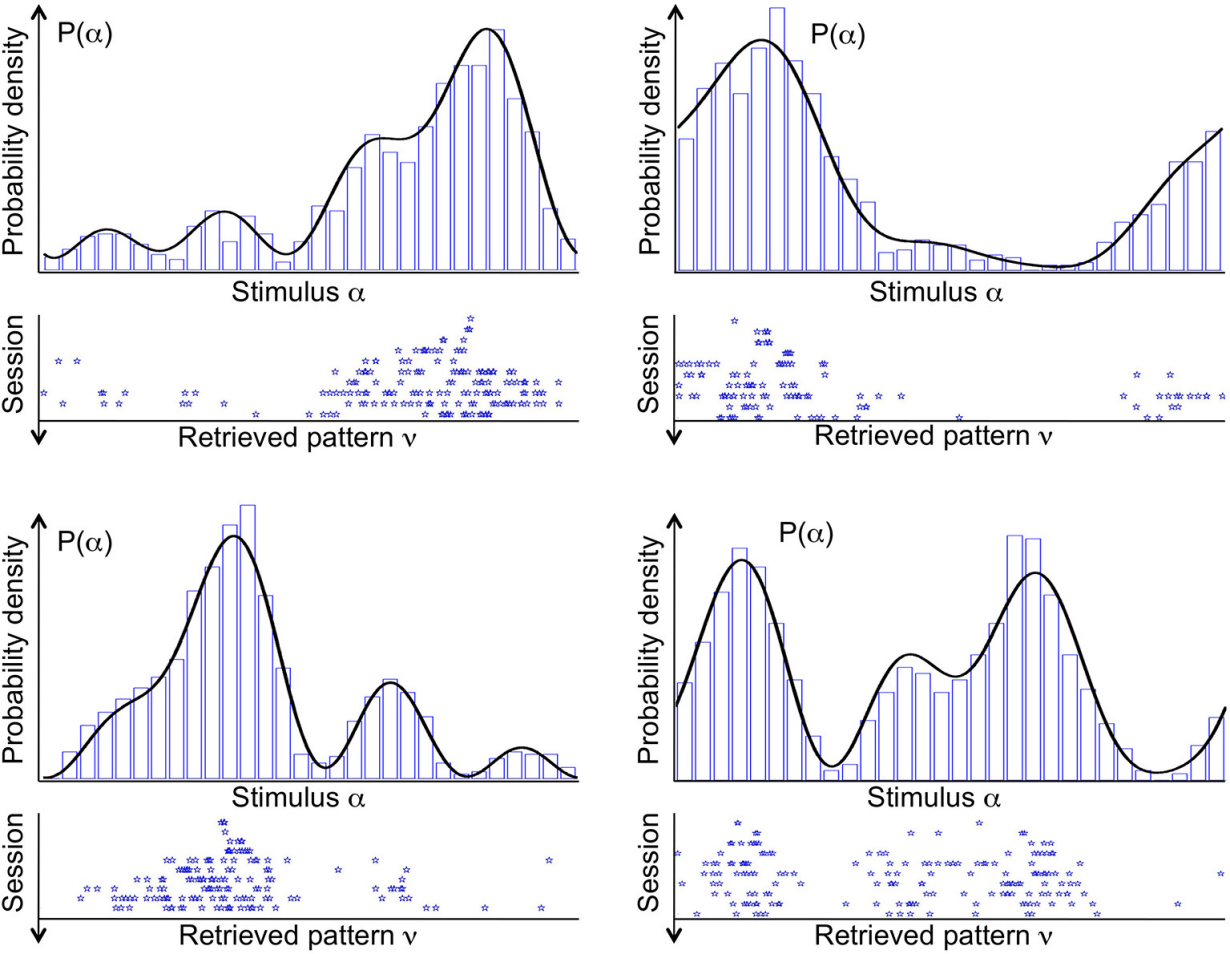
**Distribution of stimuli *p*(α) (probability density, black curve) from which the sequence of input stimuli is drawn, and the stable fixed points of the spontaneous dynamics (attractors, blue stars), referred to as “retrieved patterns.”** Four example simulations are shown in the four panels. Each stable fixed point is denoted by a star along the stimulus space. Different rows in each panel correspond to the 10 sessions of that simulation, ordered from top to bottom. In early sessions, a few attractors tend to locate near the modes of the probability density. In late sessions, several attractors sample the entire space of stimuli in proportion to the their likelihood. A histogram of the attractors from 1000 sessions (blue bars) is supermposed to the probability density of stimuli.

Figure 3 shows the stable states (retrieved patterns), recorded during 10 subsequent sessions of spontaneous dynamics (ten rows, from top to bottom), plotted together with the distribution of input stimuli (top curve), in four different simulations (four panels). In each simulation, a different probability density *p*(α) is used to draw the sequence of input stimuli. Stimuli that are located near the highest mode of the distribution are more likely to be presented. Therefore, the corresponding neural activity patterns occur more often, and drive synaptic plasticity toward their own stabilization. As a consequence, in the first session one attractor state appears near the highest mode of the distribution (top row). In one simulation, two attractors appear near the highest mode. In another simulation, two attractor states appear, one near the highest mode, and another one near the second highest.

The landscape of attractor states changes significantly in ten subsequent sessions (Figure 3, ten rows, from top to bottom). First of all, attractor states are not maintained. If a given state is an attractor in one session, it is not necessarily an attractor in the next session. This is a consequence of the ongoing synaptic plasticity and the ongoing presentation of stimuli. Both processes are noisy: synaptic transitions are stochastic, and stimuli are drawn at random from the given probability density *p*(α). Most importantly, the number of attractor states increases significantly in subsequent sessions. In each simulation, the first session has only one or at most two attractor states. Numerous attractor states appear in subsequent sessions, which seems to sample precisely the distribution of input stimuli.

In summary, spontaneous activity of the neural circuit shows a large number of stable states which samples exactly the distribution of input stimuli. Therefore, spontaneous activity tends to linger on neural activity patterns that corresponds to specific input stimuli, more so if those stimuli have been experienced more often. Formally, spontaneous neural activity stops at a stable state and stays there indefinitely. However, in presence of noise, spontaneous activity would jump between attractor states (Amit, 1992), and would spend more time where a larger number of attractor states are present. In addition, I show below that an infinite number of stable fixed points (a continuous attractor) develops in the limit of an infinite number of neurons, implying that spontaneous activity is virtually free to sample the distribution. This property makes the neural circuit akin to a generative model of the stimuli (see Discussion).

**Figure 4:**
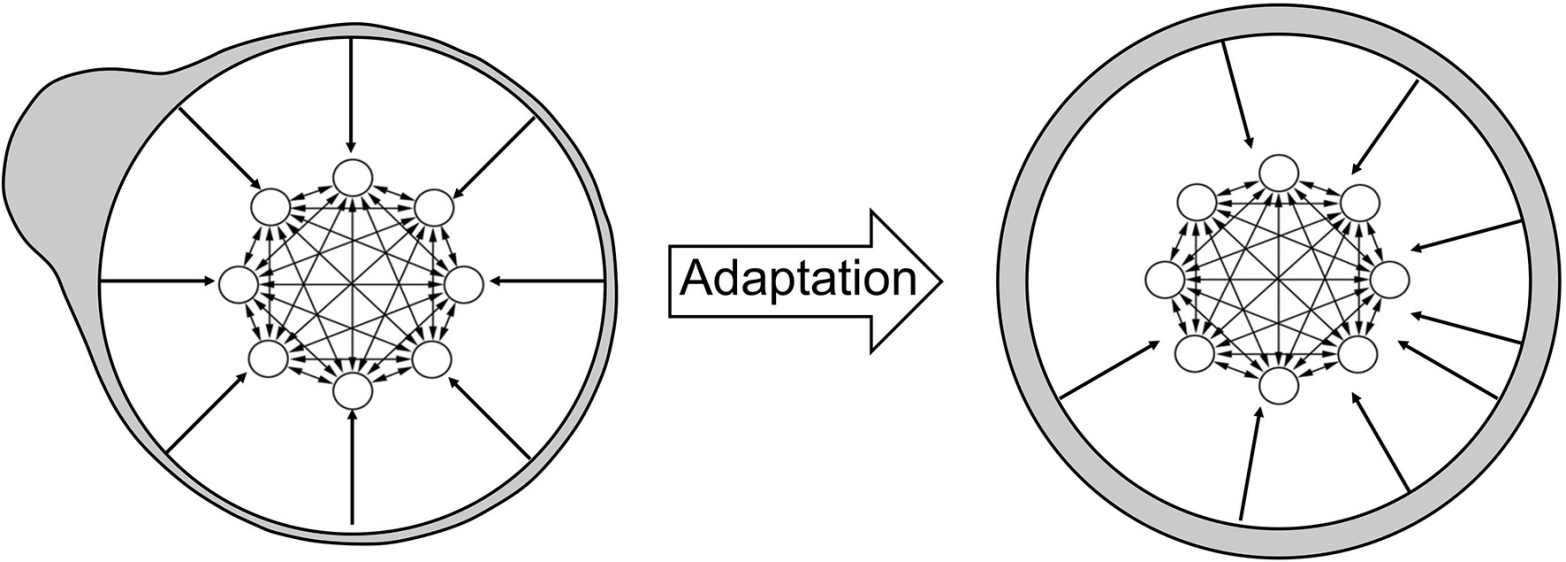
**Effect of adaptation on the representation of stimuli**. **Left**: illustration of the neural circuit model, same as in Figure 1. The tuning curves of different neurons are not shown here, but are still represented by the arrows pointing from the external circle to the neural circuit. The gray shading illustrates the distribution of external stimuli according to the network selectivity: the bump on the top left of the figure implies that most stimuli are presented in that region. By definition, a stimulus presented at a given place of the neural circuit is intended as equal to the tuning offset of the corresponding neuron. **Right**: after adaptation, the tuning curves of neurons are changed, as shown by the displacement of the arrows from the bulk of the stimulus distribution. As a consequence, the stimulus distribution in gray shading now appears uniform across the network (uniform gray shading around the circle).

The increase in the number of attractor states is a consequence of adaptation, as illustrated in Figure 4. Initially, synapses tend to favor neural activity patterns of stimuli that are encountered more often. However, adaptation tends to counterbalance this effect. In order to illustrate this, I associate each stimulus with a specific region of the neural circuit: when a stimulus is equal to the tuning offset of a neuron, I associate the stimulus with the location of that neuron. In Figure 4 (left), most stimuli are presented (gray shading) in the top left part of the network (therefore, most stimuli are equal to the tuning offsets of neurons in that part). As a consequence of adaptation, tuning offsets of neurons also tend to concentrate in that part of the network. This is illustrated by the external arrows that, representing fixed shapes of tuning curves, are “repelled” by those stimuli (right). The new organization of neurons following this transformation implies that the distribution of input stimuli now looks uniform (gray shading). I show in the Appendix that the distribution of tuning offsets of neurons matches exactly the distribution of presented stimuli (see Appendix: “The dynamics of tuning offsets”).

Therefore, the network effectively “sees” a uniform distribution of presented stimuli. When synaptic plasticity applies to this uniform distribution, no specific stimulus pattern is favored with respect to any other. Therefore, the distribution of synaptic strengths do not favor any specific stimulus, and all patterns are equally likely to represent an attractor state. The increase in the number of attractors across sessions reflects the fact that synaptic plasticity tends to make more and more patterns suitable for stability. However, due to the finite size of the network, the stochastic synaptic transitions and the random presentation of stimuli, some neural patterns are still more likely than others to stabilize. Note that attractors distribute uniformly in the neural space, but since the neural representation of stimuli has changed, via the change in tuning curves, the attractors follows the distribution of stimuli in the stimulus space (see Discussion) as shown in Figure 3.

**Figure 5:**
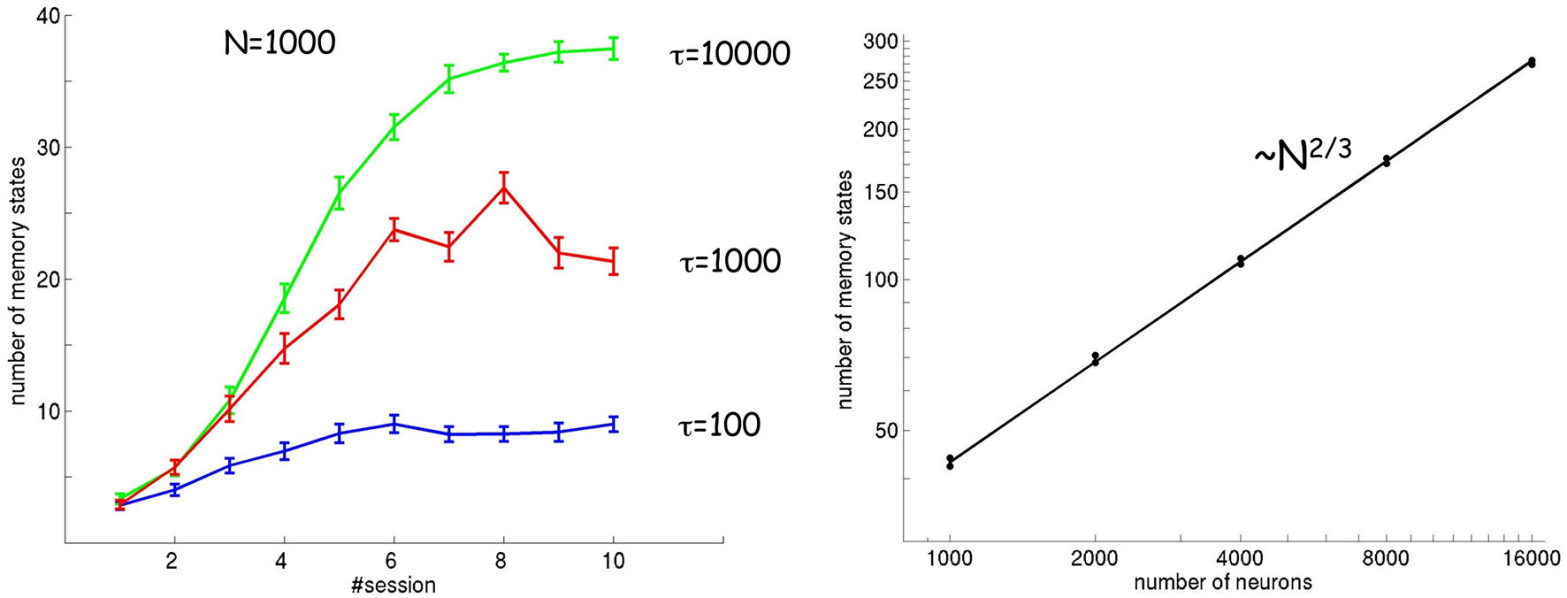
**Number of attractor states as a function of session number, timescale τ (left), and number of neurons (right)**. The number of attractor states increases in subsequent sessions and for slower timescales. The scaling of the number of attractors with respect to the number of neurons is ~*N*^2/3^.

Figure 5 (left) shows the number of attractor states as a function of session for three different values of the timescale τ of plasticity and adaptation (both phenomena are assumed to evolve according to the same time constant τ). As described above, the number of attractor states increases in subsequent sessions. In addition, the number of attractor states also increases as a function of the timescale τ. A larger timescale implies a smaller effect of noise, because changes in synaptic strengths and tuning curves are slow enough to encompass a large number of stimulus presentations and average out the resulting fluctuations.

In order for the spontaneous activity to match exactly the distribution of input stimuli, the landscape of attractor states should converge to an infinite number of fixed points (a continuous attractor) in the limit of a large number of neurons. I tested this hypothesis by looking at how the number of attractor states scales with the number of neurons. The result is shown in Figure 5 (right), where the number of attractor states is calculated in the limit of large τ and in stationary conditions. The number of attractor states increases as a function of the number of neurons according to a power law ~*N*^2/3^. Using mathematical arguments, I show in the Appendix that in the limit of large *N* and τ, the dynamics indeed converges to a continuous (line) attractor state (see Appendix: “The spontaneous dynamics of neurons”).

## 4. Discussion

It is well known that Hebbian synaptic plasticity determines stable and autonomous neural patterns of activity, sometimes called “cell assemblies,” or “attractors” (Hopfield, 1982; Bernacchia and Amit, 2007). These stable states are spontaneous, since they can activate in absence of an external stimulus. In this work, I showed that if sensory adaptation is added to synaptic plasticity, these spontaneous states replicate the activity evoked by the previously experienced stimuli, in proportion to their relative occurrence. In other words, this set of stable states samples precisely the distribution of stimuli, and the neural circuit represents a generative model of the input stimuli (Hinton, 2007, 2010; Fiser et al., 2010; Barra et al., 2012; Clark, 2013). This is consistent with the observation that spontaneous activity of neurons in visual cortex reproduces the stimulus-evoked activity (Kenet et al., 2003; Berkes et al., 2011). According to Bayesian models, neural activity may represent the prior distribution of stimuli, either by encoding the value of the probability (Pouget et al., 2003), or by sampling that distribution (Hoyer and Hyvarinen, 2003). The present work is more consistent with the latter interpretation.

Bayesian models have been applied to a broad variety of problems in Neuroscience (Vilares and Kording, 2011), including multi-sensory integration (Ernst and Bülthoff, 2004; Knill and Pouget, 2004), sensory-motor control and action selection (Körding and Wolpert, 2006; Berniker et al., 2011). Bayesian models propose that neural circuits maintain a representation of the probability distribution of sensory stimuli (prior), and combine this prior distribution with new incoming information (Fiser et al., 2010). Probability distributions are believed to be represented by the activity of populations of neurons (Pouget et al., 2003). However, while the neural mechanisms of multi-sensory integration are starting to be elucidated (Stein and Stanford, 2008; Angelaki et al., 2009), it remains unknown how the brain forms priors and how it combines them with new information (Vilares and Kording, 2011).

The model studied in this work is characterized by binary neurons and binary synapses, and includes a simple model of sensory adaptation and synaptic plasticity. Because of its simplicity, the model does not account for a range of biological phenomena observed in real neurons and synapses, and any comparison between the model and experimental data may be only qualitative. However, the model can be easily simulated and analyzed, and the results can be understood in a formal mathematical framework. Details of the mathematical analysis of the model are developed in the Appendix. It remains to be tested whether the qualitative conclusions afforded by the model may be generalized to biologically more realistic situations.

A couple of groups studied more realistic neural circuit models including synaptic plasticity and spike-frequency adaptation, and showed that they optimize information transmission (Hennequin et al., 2010), and reproduce visual responses (Zylberberg et al., 2011). However, spike-frequency adaptation is different from the adaptation studied in this work, which is usually referred to as “sensory adaptation” (Wark et al., 2007; Gutkin and Zeldenrust, 2014). Sensory adaptation is a more general phenomenon, and spike-frequency adaptation is one of several possible mechanisms by which it is implemented in neural systems. In this work, I consider sensory adaptation without referring to any specific biological mechanism. This is expressed as a change in the tuning curve of neurons according to the adapting stimulus. In particular, I consider the attraction of the tuning curve by the adapting stimulus, which has been ubiquitously observed in the case of monotonic tuning curves (e.g., sigmoidal, Kohn, 2007; Rieke and Rudd, 2009). In case of unimodal tuning curves (e.g., sine), both repulsion (Müller et al., 1999; Dragoi et al., 2000) and attraction (Kohn and Movshon, 2004) of the tuning curve by the adapting stimulus has been observed. However, note that repulsion and attraction in those cases is meant with respect to the “preferred stimulus” of a neuron, instead of the “tuning offset.” In the present model, both repulsion and attraction can be observed with respect to the preferred stimulus (see e.g., Figure 2).

A substantial assumption of this work is that the representation of the stimulus follows the change in the tuning curves of neurons. In other words, a given neural activity pattern that represents a given stimulus at some moment in time, may represent a different stimulus later, because tuning curves of neurons have changed. In other words, I assume that the “homunculus” is “aware” of adaptation, while perceptual changes seem to be consistent with an “unaware” homunculus (Seriès et al., 2009), leading to what has been previously referred to as a decoding ambiguity (Fairhall et al., 2001), or coding catastrophe (Schwartz et al., 2007). However, behavioral and physiological observations are also consistent with a homunculus that is initially unaware of adaptation, but slowly catches up after enough time has passed since changes in stimulus encoding. In the present work, this could be modeled by using a faster timescale for adaptation and a slower timescale for plasticity. Future work will investigate the effects of changes in those timescales on the network dynamics and the attractor landscape (Chaudhuri et al., 2014).

In the present model, spontaneous activity converges to an attractor, a stable state of the neural dynamics and, by definition, it stays there indefinitely. However, in presence of noise, neural activity jumps between attractors (Amit, 1992), and the dynamics visits the different attractor states equally often. Furthermore, I showed that in the limit of an infinite number of neurons, the set of attractor states becomes infinite and converges to a continuous (line) attractor spanning the entire stimulus set. In that limit, the dynamics of neurons would not display discrete jumps, rather it would sample exactly and uniformly the continuous space of attractors.

As a final remark, note that in addition to representing a generative model of input stimuli, the model described here represents a solution to the problem of developing a continuous attractor from a set of discrete attractors, as previously investigated by Koulakov et al. (2002), Renart et al. (2003), Blumenfeld et al. (2006), Itskov et al. (2011).

### Conflict of interest statement

The authors declare that the research was conducted in the absence of any commercial or financial relationships that could be construed as a potential conflict of interest.

## Appendix

In this section, I derive useful expressions of the following quantities: (1) The stationary distribution of synaptic strengths; (2) The stationary distribution of tuning offsets; (3) The stable states of the spontaneous dynamics. Those quantities are instrumental for understanding the results presented in the main text.

### The dynamics of synaptic strengths

Synaptic plasticity occurs during the presentation of input stimuli, and the statistics of those stimuli affect the synaptic strengths. The goal of this section is to derive a simple expression for the stationary average of synaptic strengths, as a function of the statistics of input stimuli.

The probability that at time *t* a synapse is potentiated (*J_ij_*(*t*) = +1) is denoted by *u*(*t*). Conversely, the probability that the synapse is depressed (*J_ij_*(*t*) = −1) is denoted by *v*(*t*). The normalization condition holds, namely

(A1) u(t)+v(t)=1

Using these probabilities, the average synaptic strength is equal to

(A2) $${\langle J_{ij}\rangle }_{t}=u(t)-v(t)$$

The angular brackets denote an average over the distribution of synaptic stenghts. Using Equations (A1), (A2), the probabilities *u*(*t*), *v*(*t*) are written in terms of the average, i.e.,

(A3) $$u(t)=\frac{1+{\langle J_{ij}\rangle }_{t}}{2}$$

(A4) $$v(t)=\frac{1-{\langle J_{ij}\rangle }_{t}}{2}$$

In order to derive a master equation which describes the evolution of the average synaptic matrix 〈*J_ij_*〉, transition probabilities must be defined. The probability of potentiation, namely the probability of the transition from *J_ij_*(*t*) = −1 to *J_ij_*(*t* + Δ*t*) = +1 is denoted as *w*_+_. Conversely, the probability of depression, namely the probability of the transition from *J_ij_*(*t*) = +1 to *J_ij_*(*t* + Δ*t*) = −1 is denoted as *w*_−_. The evolution of *u*(*t*) is given by the master equation

(A5) $$u(t+\Delta t)-u(t)=w_{+}v(t)-w_{-}u(t)$$

In words, the change in the fraction of potentiated synapses during a time step Δ*t* is equal to the fraction of depressed synapses that are potentiated minus the fraction of potentiated synapses that are depressed in that time step. Substituting Equations (A3), (A4) into Equation (A5), it is possible to write the equation in terms of the average synaptic efficacy, namely

(A6) $${\langle J_{ij}\rangle }_{t+\Delta t}-{\langle J_{ij}\rangle }_{t}=-(w_{+}+w_{-})\;{\langle J_{ij}\rangle }_{t}+(w_{+}-w_{-})$$

The transition probabilities depend on neural activity according to a Hebbian prescription, given by Equations (6), (7). Substituting those equations, the above Equation (A6) is rewritten as

(A7) $$\tau \left[{\langle J_{ij}\rangle }_{t+\Delta t}-{\langle J_{ij}\rangle }_{t}\right]=-{\langle J_{ij}\rangle }_{t}+x_{i}(t)x_{j}(t)$$

This equation shows how the average synaptic strength changes according to neural activity of the pre and post-synaptic neurons. For simplicity, in the following I use Δ*t* = 1. The solution of Equation (A7) is equal to

(A8) $${\langle J_{ij}\rangle }_{t}\text{​}=\text{​}\frac{1}{\tau }\sum_{t^{\prime }\;=\;0}^{t-1}{\left(1-\frac{1}{\tau }\right)}^{t-t^{\prime }-1}x_{i}(t^{\prime })x_{j}(t^{\prime })+{\left(1-\frac{1}{\tau }\right)}^{t}{\langle J_{ij}\rangle }_{0}$$

In the following, I assume that time *t* is large enough for the initial condition to decay away, namely *t* ≫ τ. Plasticity occurs during stimulus presentation, which imposes a specific pattern of neural activity. During presentation of stimulus α(*t*) at time *t*, neural activity is equal to

(A9) $$x_{i}(t)=\xi (\mu_{i},\alpha (t))$$

Substituting Equation (A9) into Equation (A8), the expression of the average synaptic strengths is rewritten as

(A10) $${\langle J_{ij}\rangle }_{t}=\frac{1}{\tau }\sum_{t^{\prime }=0}^{t-1}{\left(1-\frac{1}{\tau }\right)}^{t-t^{\prime }-1}\xi (\mu_{i},\alpha (t^{\prime }))\;\xi (\mu_{j},\alpha (t^{\prime }))$$

As explained in the Materials and Methods section, in each trial one stimulus α is drawn at random from a distribution *p*(α). In the limit of large *t* and τ (and *t* ≫ τ), the sum over *t*′ in Equation (A10) can be replaced by an integral over the distribution of the stimulus values, and the stationary synaptic strengths are equal to

(A11) $$\langle J_{ij}\rangle =\int_{0}^{1}d\alpha \;p(\alpha )\xi (\mu_{i},\alpha )\xi (\mu_{j},\alpha )$$

### The dynamics of tuning offsets

In addition to plasticity, during the presentation of input stimuli also adaptation takes place, and determines a change in the tuning offsets of neurons. The goal of this section is to derive a simple expression for the distribution of tuning offsets as a function of the statistics of input stimuli. The final result is that, under certain assumptions, the distribution of tuning offsets is equal to the distribution of input stimuli.

The dynamics of tuning offsets is defined by Equation (8). That equation is rewritten here with a minor rearrangement of terms

(A12) $$\tau [\mu_{i}(t+\Delta t)-\mu_{i}(t)]=\mu_{i}^{0}-\Theta (\mu_{i}(t)-\alpha (t))$$

I assume that after enough time has passed, this dynamics settles into a stationary state. In particular, this assumption will be met in the limit of large τ. The stationary state is defined by the condition that the temporal average of μ_*i*_(*t* + Δ*t*) is equal to the temporal average of μ_*i*_(*t*). In other words, the temporal average of the left hand side is zero. Therefore, the temporal average of the right hand side must be also zero. The temporal average of the Θ step function is equal to the fraction of times in which its argument is positive, namely the probability that μ_*i*_ > α. On the other hand, μ^0^_*i*_ is constant and given by Equation (9). Therefore, the stationary condition reads

(A13) $$\text{Prob}(\mu_{i}>\alpha )=\mu_{i}^{0}=\frac{i-1/2}{N}$$

Note that μ^0^_*i*_ represents an ordered and uniform tiling of the interval (0,1) by neurons. At the stationary state, when the values of μ_*i*_ are relatively fixed and stable, the left hand side is equal to the cumulative distribution function of the input stimuli α. I denote the density and cumulative distribution function of the input stimuli by, respectively, *p*(α) and *P*(α). Therefore, Equation (A13) is rewritten as

(A14) $$P(\mu_{i})=\mu_{i}^{0}$$

By definition of cumulative distribution function, *P* can be inverted to obtain the tuning offsets of neurons

(A15) $$\mu_{i}=P^{-1}(\mu_{i}^{0})$$

In the limit of large *N*, I can substitute the index *i* with a label *y* = μ^0^_*i*_ spanning a continuum of neurons in the interval (0,1). Therefore,

(A16) $$\mu (y)=P^{-1}(y)$$

Then, it is easy to show that the tuning offsets of neurons follow the same distribution of input stimuli. By taking the derivative of Equation (A16) with respect to *y*, I can calculate the volume *dy* of neurons with tuning offsets included in a given interval *d*μ, that is equal to

(A17) dy=p(μ)dμ

Therefore, the density function of tuning offsets across neurons is equal to the *p*(μ), which is equal to the density of input stimuli. I denote the density of tuning offsets as *g*(μ) and the density of input stimuli as *p*(α). Therefore, the above result is written as

(A18) g(μ)=p(μ)

### The spontaneous dynamics of neurons

The goal of this section is to derive a simple equation describing the (stable) fixed points of the retrieval dynamics of neurons, that is the dynamics in absence of stimulus and plasticity. However, I assume that stimulation and plasticity have occurred before this retrieval stage, and synaptic strengths have been modified by the distribution of input stimuli through Equation (A11). Therefore, the distribution of input stimuli in turn affects the neural dynamics during retrieval. Neural dynamics also depends on the distribution of tuning offsets.

During the retrieval phase there is no input stimulus, therefore the total current *I_i_* is equal to the internal current, given by Equation (5). In the limit of a large number of neurons *N*, I can substitute the synaptic strength *J_ij_* with its average, therefore the current is equal to

(A19) $$I_{i}(t)=\frac{1}{2N}\sum_{j}\langle J_{ij}\rangle \;x_{j}(t)$$

I substitute the expression for the average synaptic strength, Equation (A11), into Equation (A19), and the new expression for the current is

(A20) $$I_{i}(t)=\frac{1}{2N}\int_{0}^{1}d\alpha \;p(\alpha )\xi (\mu_{i},\alpha )\sum_{j\;=\;1}^{N}\xi (\mu_{j},\alpha )x_{j}(t)$$

As mentioned above, during the retrieval phase there is no input stimulus. Nevertheless, I assume that neural activity is equal to a pattern that would be obtained by stimulating the network with a given stimulus ν. Namely, I assume that

(A21) $$x_{j}(t)=\xi (\mu_{j},\nu (t))$$

The value of ν depends on time and is unknown. This assumption is verified below by a self-consistent argument. Under this assumption, the new expression of the current is obtained by substituting Equation (A21) into Equation (A20), to find

(A22) $$I_{i}=\frac{1}{2N}\int_{0}^{1}d\alpha \;p(\alpha )\xi (\mu_{i},\alpha )\sum_{j\;=\;1}^{N}\xi (\mu_{j},\alpha )\xi (\mu_{j},\nu )$$

where I dropped the explicit dependence on time. In the limit of a large number of neurons *N*, I can substitute the sum over the index *j* with an integral over the tuning offsets of neurons, μ = μ_*j*_, according to the distribution of such tuning offsets, which I denote by *g*(μ). Namely, I can use the following expression

(A23) $$\frac{1}{N}\sum_{j=1}^{N}\xi (\mu_{j},\alpha )\xi (\mu_{j},\nu )=\int_{0}^{1}d\mu \;g(\mu )\xi (\mu ,\alpha )\xi (\mu ,\nu )$$

By substituting Equation (A23) into Equation (A22), the new expression for the current is

(A24) $$I_{i}=\frac{1}{2}\int_{0}^{1}d\alpha \;d\mu \;p(\alpha )g(\mu )\xi (\mu_{i},\alpha )\xi (\mu ,\alpha )\xi (\mu ,\nu )$$

This expression is useful for studying the stable states of neural dynamics. However, first the assumption made in Equation (A21) must be checked. The following analysis is valid in case of a sigmoidal tuning curve, Equation (3), which implies ξ(μ, ν) = sign(μ − ν), but similar results are obtained in case of a periodic tuning curve Equation (4).

The assumption in Equation (A21) states that neural activity in absence of the stimulus is equal to the activity in presence of a stimulus, for a given unknown stimulus ν at time *t*. In order to check this assumption, I follow a self-consistent argument: given that the assumption is true at time *t*, test whether it remains true at time *t* + Δ*t*. In other words, given Equation (A21), it must be true that

(A25) $$x_{i}(t+\Delta t)=\xi (\mu_{i},\nu (t+\Delta t))$$

Note that the ν(*t* + Δ*t*) is not given and can be any value in the interval (0,1). Equation (A24) was derived under the assumption of Equation (A21), therefore I check whether Equation (A24) implies Equation (A25). I substitute the expression for the function ξ, Equation (10) in Equation (A24). After calculating the integral explicitly, I express the current in terms of the cumulative distribution functions of the input stimuli *P* and tuning offsets *G*, whose density functions are, respectively, *p* and *g*. The result is

$$\begin{array}{l} I_{i}=G(\nu )-P(\mu_{i})+\frac{1}{2}\int_{0}^{1}d\alpha [P(\alpha )g(\alpha )-p(\alpha )G(\alpha )]+\text{sign}(\mu_{i}-\nu )\cdot \\ \cdot \left\{\left[P(\mu_{i})-P(\nu )][G(\mu_{i})-G(\nu )]+[P(\nu )G(\mu_{i})-P(\mu_{i})G(\nu )]-\int_{\nu }^{\mu_{i}}d\alpha [p(\alpha )G(\alpha )-P(\alpha )g(\alpha )\right]\right\} \end{array}$$

Note that the term in curly brackets is small for μ_*i*_ ~ ν. In that case, the current is approximated by

(A26) $$I_{i}≃G(\nu )-P(\mu_{i})+\frac{1}{2}\int_{0}^{1}d\alpha [P(\alpha )g(\alpha )-p(\alpha )G(\alpha )]$$

This is a monotonically decreasing function of μ_*i*_, because the cumulative distribution *P* is monotonically increasing by definition. In addition, the current *I_i_* takes opposite values at the extreme points μ_*i*_ = 0 and μ_*i*_ = 1. Therefore, it must vanish at a unique value of μ_*i*_, provided that this value is close enough to ν to justify the approximation μ*_i_* ~ ν. I define this value as ν(*t* + Δ*t*). As a consequence, using Equation (2), the neural activity is equal to

(A27) $$x_{i}(t+\Delta t)=\text{sign}(I_{i})=\text{sign}(\nu (t+\Delta t)-\mu_{i})=\xi (\mu_{i},\nu (t+\Delta t))$$

and Equation (A25) is demonstrated. Therefore, the assumption that neural activity in absence of the stimulus takes the form of Equation (A21) is self-consistent. The value of ν(*t* + Δ*t*) can be calculated as a function of the value of ν(*t*). Under the assumption that the two values are close, ν(*t* + Δ*t*) ~ ν(*t*), this can be calculated by replacing μ_*i*_ with ν(*t* + Δ*t*) in Equation (A26). Therefore,

(A28) $$P(\nu (t+\Delta t))-G(\nu (t))+\frac{1}{2}\int_{0}^{1}d\alpha [p(\alpha )G(\alpha )-P(\alpha )g(\alpha )]=0$$

In order to find the fixed points of neural activity, I impose that ν(*t* + Δ*t*) = ν(*t*) = ν. The value of ν is the fixed point of the retrieval dynamics. This condition is equivalent to

(A29) $$P(\nu )-G(\nu )+\frac{1}{2}\int_{0}^{1}d\alpha [p(\alpha )G(\alpha )-P(\alpha )g(\alpha )]=0$$

This equation can be solved to find the fixed points ν for a given distribution of input stimuli *p* and of tuning offsets *g*. By taking the derivative of Equation (A28) with respect to ν, it is easy to check that a fixed point ν is stable if and only if

(A30) p(ν)>g(ν)

Note that if the two distributions *p* and *g* are exactly equal, as happens at the stationary state of the dynamics of tuning offsets, Equation (A18), then all possible values of ν correspond to marginally stable fixed points. This corresponds to a continuous attractor state.
